# Bidirectional Long Short-Term Memory Model of *SoH* Prediction for Gelled-Electrolyte Batteries under Charging Conditions

**DOI:** 10.3390/gels9120989

**Published:** 2023-12-17

**Authors:** Ting-Jung Kuo, Wei-Ting Chao

**Affiliations:** 1 Department of Applied Artificial Intelligence, Ming Chuan University, Taoyuan 33348, Taiwan; tingjung@mail.mcu.edu.tw; 2 Center of Excellence for Ocean Engineering, National Taiwan Ocean University, Keelung 20224, Taiwan

**Keywords:** gelled-electrolyte battery, bidirectional long short-term memory, state of health

## Abstract

The impact of different charging currents and surrounding temperatures has always been an important aspect of battery lifetime for various electric vehicles and energy storage equipment. This paper proposes a bidirectional long short-term memory model to quantify these impacts on the aging of gel batteries and calculate their state of health. The training data set of the bidirectional long short-term memory model is collected by charging and discharging the gel battery for 300 cycles in a temperature-controlled box and an automated charge and discharge device under different operating conditions. The testing set is generated by a small energy storage device equipped with small solar panels. Data for 220 cycles at different temperatures and charging currents were collected during the experiment. The results show that the mean absolute error (MAE) and root-mean-square error (RMSE) between the training set and testing set are 0.0133 and 0.0251, respectively. In addition to the proposed model providing high accuracy, the gel battery proved to be stable and long-lasting, which makes the gel battery an ideal energy storage solution for renewable energy.

## 1. Introduction

The valve-regulated lead-acid (VRLA) battery is one of the most frequently used types of rechargeable batteries. Due to low material costs, robustness, high reliability, and the ability to instantaneously discharge large currents, the VRLA is widely used in various applications, including light electric vehicles, backup power systems, and ignition systems. VRLA battery technology has undergone numerous improvements and refinements, focusing on enhancing its performance and extending its lifetime. Electrodes, separators, and electrolytes are the main components of the VRLA system. Each component is crucial to the performance and lifetime of a battery. For the electrolyte component, researchers have created technologies such as absorbent glass mats (AGM) and gelled electrolytes that use gel instead of liquid acid as the electrolyte [1]. Pyrogenic silica is a highly pure amorphous silicon dioxide capable of absorbing more than ten times its weight in acidic liquids to form a gel [2,3]. The gelled electrolytes are usually made by uniformly dispersing fumed silica in sulfuric acid [4]. The unique properties of the gel allow it to connect molecules to form a network and retain liquid, resulting in the formation of a gel structure after a certain period. Therefore, the electrolyte of the gel battery can exist in a solid state between the positive and negative plates inside the battery [5]. The advantages of gel batteries over traditional lead-acid (flooded) batteries are as follows:

- When the gel battery is charged, hydrogen gas is absorbed by the plates and converted to electrolytes. It does not require adding water or equalizing the charge, so it requires no maintenance and is very safe to use.
- The battery has a low self-discharge, instant high-power output, and long cycle life.
- The electrolyte is stable inside the battery, and the battery can be installed in any desired direction.
- At extreme temperatures, the gelled electrolyte prevents the electrolyte from evaporating or freezing and ensures high performance. If excessive gas is generated from improper charging, the safety valve automatically discharges the gas to prevent the battery from rupturing.

The heat and electrochemical polarization reaction, ohmic loss, oxygen recombination cycle, and other factors generated by a battery during the cycle will directly affect the available capacity of a battery [6]. For example, a deep-cycle discharge can cause the electrolyte to stratify quickly, decreasing the battery capacity. This is due to the irreversible degradation of the battery’s grid corrosion and the high concentration of active material in the lower regions of the plates. Since the gelled electrolyte is less stratified than AGM’s electrolyte, it can last longer during cycling, providing outstanding durability and enduring more cycling [7].

Compared to lithium-ion batteries, gel batteries have lower power and energy density. However, gel batteries are typically the most appropriate solution for light electric vehicles (such as golf carts, small boats, etc.), where the manufacturing cost and safety of the battery are essential factors. Additionally, the lead-acid battery system has been developed since 1859. It has a complete manufacturing process and recycling mechanism, and a lower environmental impact than lithium-ion batteries [8,9,10]. Therefore, it is one of the reasons why gel batteries are superior to lithium-ion batteries in terms of environmental protection.

Small solar or wind energy generators are built in urban areas and on school grounds to compensate for the energy loss caused by long-distance electricity transmission through the power grid. Renewable energy is, however, highly dependent on weather conditions due to its instability. In this regard, small battery energy storage devices are necessary. Whenever solar or wind power produces more power than the system requires, excess power is stored in the batteries When the energy produced is insufficient, the batteries power the system. Although the volume-to-capacity ratio of the gel battery is lower than that of AGM, the gel battery can provide reliable, maintenance-free power in many deep-cycle applications, such as solar energy and light service vehicles [11,12].

The above applications have heightened interest in gel batteries’ charging rate and service life. Reducing the charging time is crucial to increasing people’s acceptance of renewable energy. Fast charging usually involves a high current, possibly accelerating battery degradation [13,14,15]. Fast charging is also at odds with extending the gel battery life and lowering maintenance costs. Therefore, it is necessary to determine the aging mechanism of gel batteries and quantify the impact of different charging stresses on the battery to find the optimal charging mechanism.

An optimal battery charging mode balances charging speed and battery cycle life. Studies [16,17] have reported many charging strategies to increase charging speed, enhance charging performance, and maximize battery cycle life, such as optimizing charging strategies to suppress side reactions based on the electrochemical characteristics of the battery. These strategies can reduce battery degradation but are nearly impossible to implement in real-life applications. In the research of [18,19,20], optimal charging strategies are based on equivalent circuit models to adjust the charging efficiency and cycle life. These strategies are easy to implement but need more support from electrochemical mechanisms. Researchers [21,22] have proposed a multi-level constant-current charging strategy to find the best charging mode for the battery during the charging process. Researchers [23,24] have studied the impact of multi-level fast charging on the aging process of batteries. These studies compared and evaluated different charging strategies and charging stresses, including the charging current and charging cut-off voltage, but did not discuss the performance and aging mechanism of the battery under different operating temperatures.

The charging strategies mentioned previously lack physical or chemical theorems because the battery’s aging mechanism under different charging current rates and operating temperatures is unclear. Establishing a model describing the relationship between the fading capacity and charging stress is very difficult. Researchers [25,26,27] have studied battery degradation under different operating conditions, such as the ambient temperature, state of charge (*SoC*) range, charge/discharge rate, and cut-off voltage, through which battery life can be extended. In work [28], researchers reported that to delay battery degradation, the charging process can be significantly affected by adjusting the charging current and cut-off voltage. In work [29], researchers studied the aging behavior of batteries during long-term cycling at high charging rates to explain the aging effects of batteries. In this paper, based on these reports, we propose a model for accurately estimating the state of health (*SoH*) of gel batteries under different operating conditions.

### 1.1. Contributions of the Paper

This paper conducted a cycle life test on commercial 12 Ah gel batteries to demonstrate the aging mechanism of the batteries under different charging currents. The bidirectional long short-term memory (BiLSTM) model was used to quantify and estimate the gel battery’s *SoH* to verify its characteristics. The training set is produced by an automatic charging and discharging device in the laboratory. A working small energy storage device generates the testing set. The main contributions of this paper are summarized as follows:

- There are few studies related to gel battery performance. This study intends to conduct 300 deep cycles under different charging currents and temperatures to examine the characteristics of the gel electrolyte.
- This study develops a deep-learning model of the battery cycle life to describe the relationship between the battery’s capacity fading, charging stress, and operating temperature.
- In a working environment of 25 °C, the high charging current impacts battery life but is still in a safe range. However, at a high temperature of 50 °C, in order to avoid the intense electrochemical reaction inside the battery, the charging current needs to be reduced.

### 1.2. Organization of the Paper

This paper is organized as follows: Section 2 introduces the design of the experiment, including the deep-cycle life testing of the battery under different charging currents and temperatures. The test results related to battery aging, including *SoC* and *SoH*, are also presented in this section. Section 3 analyzes the degradation mechanism of the battery and proposes a BiLSTM model to establish the *SoH* model. The data for the model comes from laboratories and a small energy storage device. Section 4 discusses the results in depth. Section 5 summarizes the characteristics of the gel battery and the performance of our proposed BiLSTM model.

## 2. Experimental Setup and Procedures

In order to verify whether the battery’s *SoH* model can improve the prediction accuracy in actual cases, we propose using BiLSTM to build a deep-learning model. The model data collection was divided into a training set and testing set. Data for the training set were collected in the laboratory using automatic battery charging and discharging devices. Data for the testing set are collected when the gel battery is connected to the solar energy generation device and the microgrid. Gel battery data were collected during the charging and discharging process based on the load, voltage, current, temperature, and time.

### 2.1. Battery Testing Set Setup

**Figure 1:**
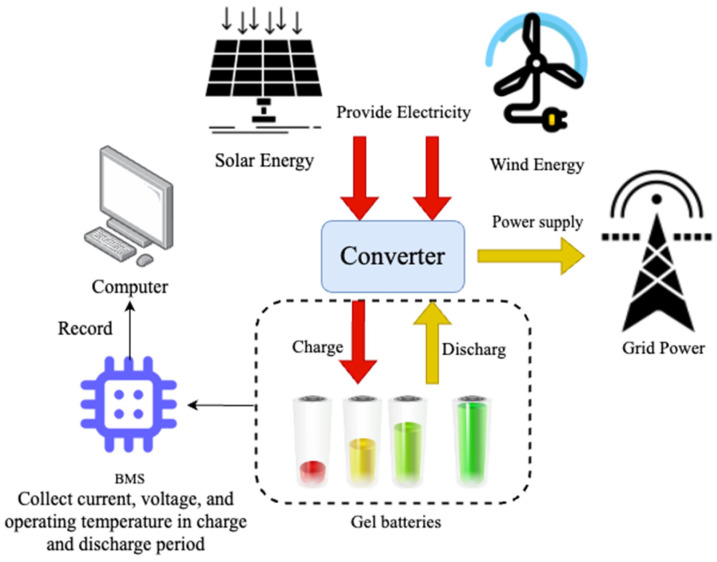
The architecture of a small demonstration energy storage center.

Figure 1 illustrates a small demonstration energy storage device built on campus. When renewable energy (such as solar or wind) is abundant, the device supplies power for the campus, and the excess power is used to charge the gel batteries. The fully charged batteries are connected to the microgrid and discharged through loads (such as low-power equipment) on the microgrid. The small demonstration energy storage device has a simple air conditioner to dissipate heat. The air conditioner is only turned on when the battery’s operating temperature is higher than 50 °C to keep the batteries within a safe operating temperature range as well as reduce power consumption from the air conditioner.

During the cycle process, a battery management system (BMS) monitors and records the battery’s operating temperature, voltage, and current. Our main goal is to study the impact of charging strategies on the battery’s cycle life under various operating conditions and to propose recommended charging strategies based on the *SoH* of the battery.

### 2.2. Gel Battery Training Set Setup

**Figure 2:**
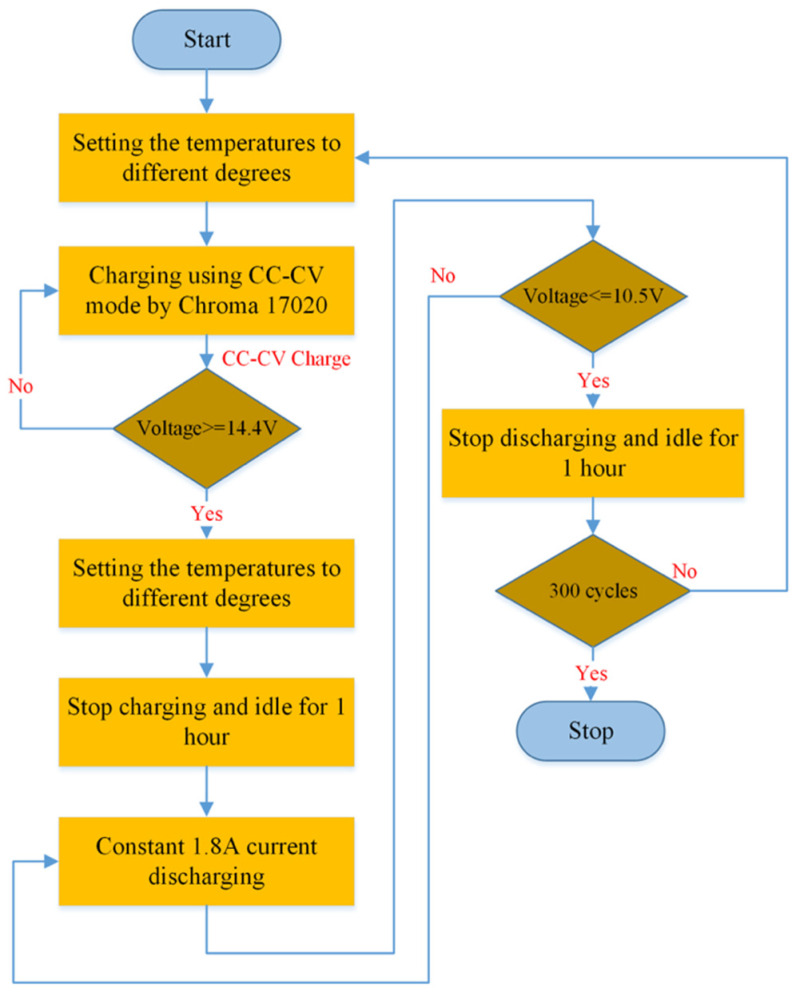
Experimental setup flowchart.

In order to study the aging mechanism of gel batteries and establish the *SoH* under different operating conditions, the batteries were charged under different currents and operating temperatures. The discharge process was executed at a fixed discharge current and temperature. It is used to evaluate the performance of gel batteries and determine their cycle life to determine the impact of varying charging currents and temperatures. The specifications of the tested gel batteries are listed in Table 1. The batteries were charged and discharged using a Chroma 17020 battery system from Taiwan’s Chroma corporation. The experimental process is shown in Figure 2.

**Table 1:** Nominal specifications of the gel battery.

Item	Specification
Nominal capacity	12 Ah
Charging method	Constant current and constant voltage (CC-CV)
Max. charging current	6 A
Charging cut-off voltage	14.4 V
Max. discharging current	6 A
Discharging cut-off voltage	10.5 V

By definition, one battery cycle consists of a complete charge and discharge at the cut-off voltage. This paper used three batteries as a group to carry out corresponding experimental conditions to ensure data consistency. New batteries were charged using the constant-current and constant-voltage (CC-CV) method with different maximum currents (1.8, 3.6, and 5.4 A) until the voltage reached 14.4 V. After each complete charge, batteries were left idle for 1 h to allow the electrochemical reaction inside the battery to reach a steady state. Then, each battery was discharged at a current of 1.8 A until the voltage dropped to the 10.5 V cut-off voltage. All the tested batteries were charged and discharged 300 times in the cycle. The experimental parameter settings are listed in Table 2.

### 2.3. Charge Curves Analysis of Batteries under Different Charge Current and Cycle Numbers at 25 °C

**Figure 3:**
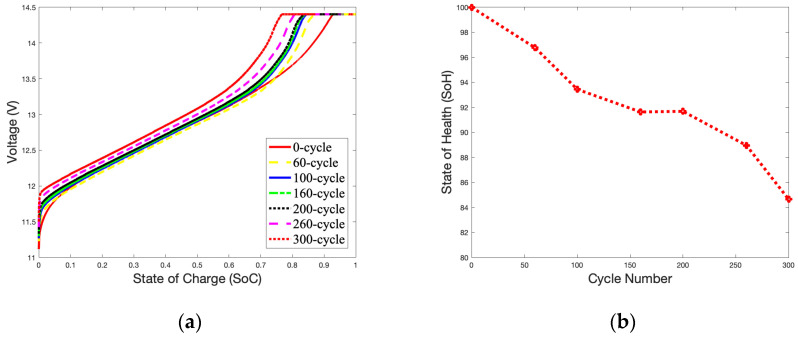
1.8 A charge *SoC* curves and *SoH* curve in 25 °C during 300 cycles: (**a**) *SoC* curves; (**b**) *SoH* curve.

The *SoC* curves of the 1.8 A current charging behavior for 60-, 100-, 160-, 260-, and 300-cycle batteries under 25 °C are illustrated in Figure 3a. The *SoC* is defined as the state of available energy in the battery. It is a reference value based on the designed rate capacity during manufacturing. The battery is fully charged at 100% and fully discharged at 0% [30], as shown in Equation (1).

(1) $${SoC}_{\left(t\right)}={SoC}_{\left(t0\right)}-\frac{1}{C_{max(t)}}\int_{t0}^{t}I_{\left(t\right)}dt$$

where *C_max_* denotes the maximum usable capacity of the battery and *I* denotes the battery current at time *t*. *Q_a_* denotes the available capacity of the battery through the multiple cycles.

The battery’s available energy will age when it is charged and discharged repeatedly. When an aging battery is fully charged, the *SoC* cannot reach 100% capacity. Thus, Equation (2) allows the *SoC* to be modified [31].

(2) $$SoC=\frac{Q_{a}}{C_{max}}$$

*Q_a_* denotes the available capacity of the battery for the charging/discharging cycle.

The *SoH* refers to either the battery’s capacity fade or to the power fade, which is defined as follows [32]:

(3) $$SoH=\frac{C_{max}}{C_{r}}\times 100\%$$

where *Cr* is the rated capacity.

The data acquired from the experiment were used as the training set for the BiLSTM model. Hence, the relationship between the variation in the capacity, energy loss, temperature, and cycle number was further investigated.

The CC-CV mode is a charging method that switches between constant-current charging and constant-voltage charging based on the battery’s current voltage. When the voltage is low, the battery is charged in the CC mode with a maximum current based on the preset. As the voltage reaches the charging cut-off voltage, the CC mode is switched to the CV mode to continue charging with a smaller current. The *SoC* curves for a battery charging in the CC mode are seen in Figure 3a when the battery starts to charge from an open-circuit voltage (OCV) to a closed-circuit voltage (CCV). The low currents used to charge the batteries indicate that they are charging in the CV mode. Compared to a brand-new battery that has not been cycled, a battery that has been cycled more times will reach the cut-off voltage more rapidly. It is evident by observing the occupied time of the CC and CV modes that as the number of cycles increases, the CC mode charging time becomes shorter, and the CV mode charging time becomes longer. As shown in Figure 3b, after 300 cycles, the *SoH* remains at 84.65%, only 13.35% below the available capacity of the brand-new battery (100%).

**Figure 4:**
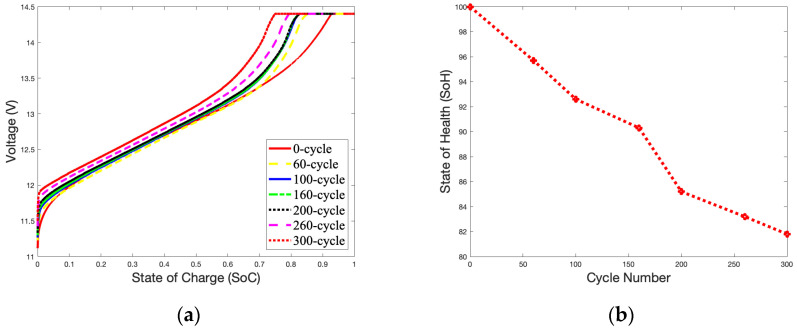
3.6 A Charge *SoC* curves and *SoH* curve in 25 °C during 300 cycles: (**a**) *SoC* curves; (**b**) *SoH* curve.

Figure 4a,b illustrate the *SoC* and *SoH* curves for gel batteries charging at 25 °C with a 3.6 A charge over 300 cycles. The battery’s *SoH* is only slightly affected by the 3.6 A charging current compared to 1.8 A. After 300 cycles, the battery’s capacity is 81.8%, only 18.2% lower than that of a brand-new battery.

**Figure 5:**
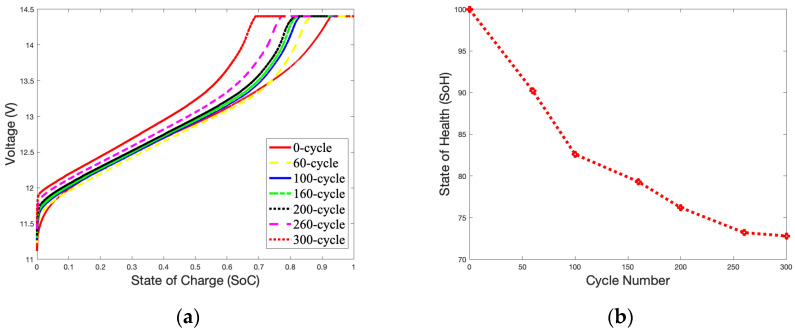
5.4 A Charge *SoC* curves and *SoH* curve in 25 °C during 300 cycles: (**a**) *SoC* curves; (**b**) *SoH* curve.

Figure 5a,b illustrate the *SoC* and *SoH* curves charging at 25 °C with 5.4 A over 300 cycles. Compared with the 1.8 A and 3.6 A charging current, the 5.4 A charging current has a higher impact on the battery cycle life. Compared with the available power capacity of a brand-new battery, it decreases by 27.2%.

### 2.4. Charge Curves Analysis of Batteries under Different Charge Current and Cycle Numbers at 50 °C

**Figure 6:**
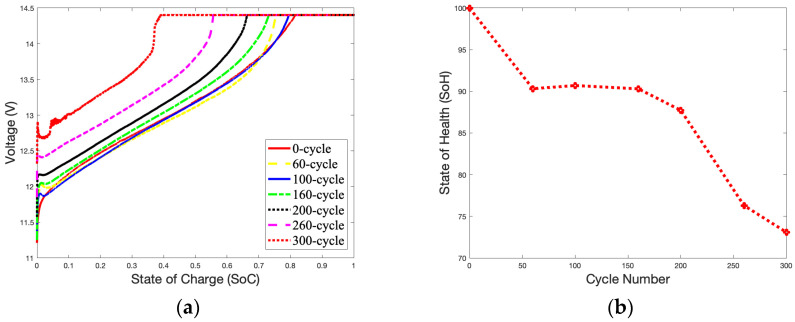
1.8 A Charge *SoC* curves and *SoH* curve in 50 °C during 300 cycles: (**a**) *SoC* curves; (**b**) *SoH* curve.

A temperature of 50 °C was selected to study the impact of surrounding temperatures on the battery’s capacity during charging. Figure 6a shows an unstable charging *SoC* curve compared with Figure 3a. A high temperature accelerates electrochemical reactions inside the battery, and the charging curve will quickly approach the cut-off voltage. When the battery reaches 300 cycles, fluctuations in the *SoC* curve can be found in the OCV-CCV stage. Figure 6b shows that as the number of charges reaches 200 cycles, the *SoH* curve drops rapidly and declines to 73.09% at 300 cycles. As shown in Figure 3b, the *SoH* of the battery is reduced by 11.56% compared to the *SoH* of the battery with 300 cycles (84.65%).

**Figure 7:**
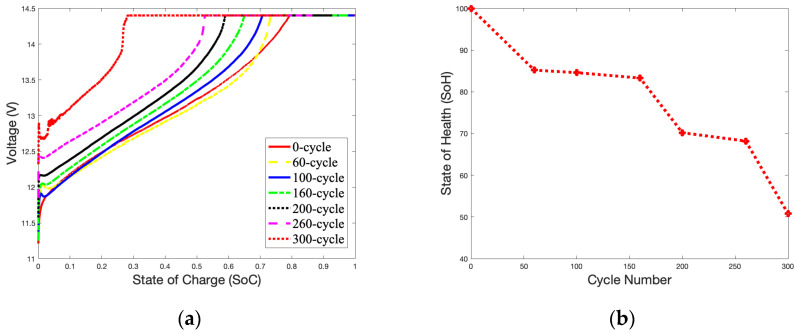
3.6 A Charge *SoC* curves and *SoH* curve in 50 °C during 300 cycles: (**a**) *SoC* of charge curves; (**b**) *SoH* curve.

Figure 7a,b illustrate the *SoC* and *SoH* curves charging at 50 °C with 3.6 A over 300 cycles. Figure 6b and Figure 7b have similar *SoH* curves. The curves from 60 cycles to 160 cycles are relatively gentle and drop rapidly after 160 cycles. Based on 300 cycles, the *SoH* of the battery is 50.8%, which is a reduction of 22.29% as compared to Figure 6b at 73.09%.

**Figure 8:**
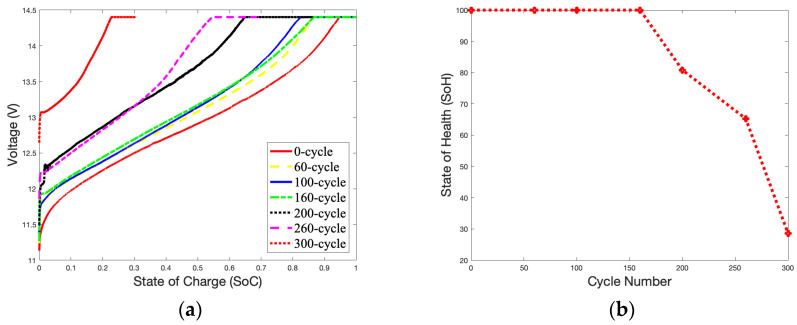
5.4 A Charge *SoC* curves and *SoH* curve in 50 °C during 300 cycles: (**a**) *SoC* curves; (**b**) *SoH* curve.

Figure 8a,b illustrate the *SoC* and *SoH* curves charging at 50 °C with 5.4 A over 300 cycles. The battery almost performed as well as a brand-new battery (*SoH* = 100%) before 160 cycles. However, the *SoH* curve suddenly dropped rapidly after 260 cycles, and reached a failure state at 300 cycles (*SoH* = 28.61%). This may be due to side effects caused by high-current charging. The thermal mechanism of the battery during charging is very complex. It is related to the electrochemical characteristics of the battery itself, such as the reaction heat, energy loss caused by battery polarization, electrolyte decomposition, self-discharge side reactions in the battery, and Joules generated by battery resistance. Heat will cause the electrochemical reaction inside the battery to be rapid due to the characteristics of the gel electrolyte. The battery’s performance was good before 160 cycled; then, the materials inside the battery began to age rapidly, causing the *SoH* of the battery to drop.

The declining trend of the *SoH* curves for the battery is partially linear, especially between 100 and 200 cycles, as show in Figure 3b and Figure 6b. At this stage, the increase in thermal mechanisms on the electrochemical reaction rate is slightly more significant than the impact of the battery’s material aging. Therefore, there is a slight increase in the battery’s capacity during this period.

The results of the above experimental analysis indicate that gelled-electrolyte batteries can be charged with a higher current (preferably not exceeding 0.45 C rate). The optimal operating temperature of the gel battery is approximately 25 °C. In this temperature range, the cycle life of the gel battery can be extended.

## 3. Bidirectional Long Short-Term Memory (BiLSTM) Model Development

Before the BiLSTM setup process, data were divided into training sets and testing sets. The training datasets consisted of all the charging characteristics for each gel battery in the laboratory. The testing datasets consisted of data on twelve 12 Ah gel batteries connected to the 48 V microgrid in the energy storage device. While the solar panels were charging the 48 V microgrid, the battery’s voltage, current, and surrounding temperature were recorded by a battery management system (BMS). The datasets collected information on the gel batteries for one year. Since rain or solar panel maintenance can affect the data, only 220 data records were usable. The acquired battery data that were used as input were battery terminal voltage (V), current (I), charge time (Ct), and operating environment temperature (T).

### 3.1. Long Short-Term Memory Architecture

**Figure 9:**
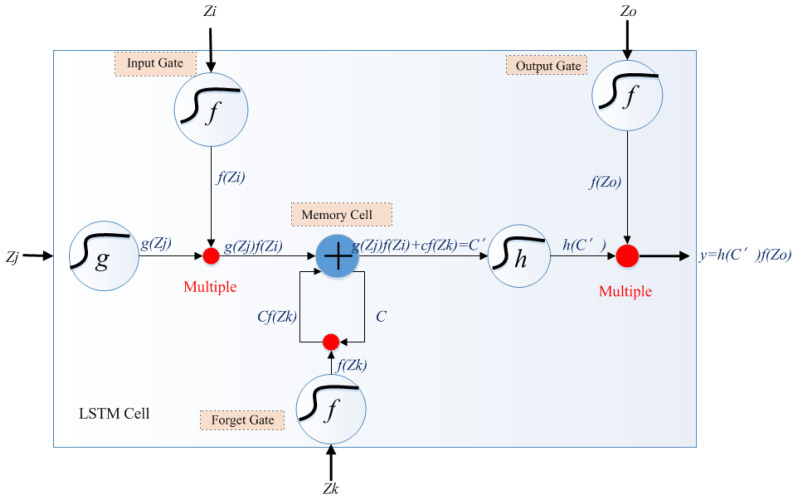
Structure of LSTM network (refer to [38]).

The long short-term memory (LSTM) model is a recurrent neural network (RNN) type designed to address the vanishing gradient problem and capture long-term dependencies on sequential data. Its architecture consists of gateways, including the forget gate, input gate, and output gate [33,34,35], as shown in Figure 9. As seen in Figure 9, the memory cell’s state is a horizontal line that runs through the LSTM unit, which acts as a memory or conveyor belt that allows information to flow through the network and preserve long-term dependencies. Its function is to act as a data bridge from data acquired in the past to currently available data [36]. When calculating the *SoH*, the capacity to recall past information makes this method especially useful in solving problems that require long sequential data or time series [37]. The LSTM cell has three gates that control the information and whether it has been updated. The operation flow of the memory cell and the function of the three gates are described as follows:

First, the initial value stored in the memory cell is called *C*. New data (*Z_j_*) inputs are multiplied by the hyperbolic activation functions *g*(*x*) to obtain the value *g*(*Zj*). The input gate regulates the flow of new information into the memory cell. After being processed by the input gate, the new data (*C^′^*) can be expressed as follows:

(4) $$C^{\prime }=g(Z_{j})f(Z_{i})$$

where *Z_i_* is the parameter to control whether the gateway is open and *f*(*x*) is a sigmoid activation function. When *f*(*Z_i_*) = 1, the memory cell is updated to 0. Otherwise, it is not updated.

The forget gate is a sigmoid layer that decides what information from the memory cell state should be forgotten or retained. It takes the previous cell state (*C*) and the current input (*Z_k_*) to produce a forget gate output between 0 and 1 for each component of the cell state. When *Cf*(*Z_k_*) is 1, then *C* is preserved; otherwise, it is canceled. Then, the memory cell state can be updated as follows:

(5) $$C^{\prime }=g\left(Z_{j}\right)f\left(Z_{i}\right)+Cf(Z_{k})$$

Then, *C*^′^ is stored in the memory cell and called *C*. Before being processed by the output gate, *C*^′^ is multiplied to a hyperbolic tangent function (*h*(*x*)) to obtain (*h*(*C*^′^)). The output gate determines the next hidden state, the filtered version of the memory cell state, and is shared with the next time step. It considers the previous cell state, the current input (*Z_o_*), and the candidate cell state (*C*^′^) to produce a value between 0 and 1. After being processed by the output gate, the output value (*y*) can be expressed as follows:

(6) $$y=h\left(C^{\prime }\right)f\left(Z_{o}\right)$$

### 3.2. Bidirectional Long Short-Term Memory Architecture

The BiLSTM model is an extension of the LSTM architecture that captures information from past and future time steps. The BiLSTM model consists of two LSTM networks, processing the input sequence in the forward and backward direction. The forward LSTM network reads the input sequence from left to right, creating hidden states at each time step. The backward LSTM network processes the input sequence in the reverse order, reading it from right to left. It also produces hidden states but captures information on the sequence in a backward manner.

**Figure 10:**
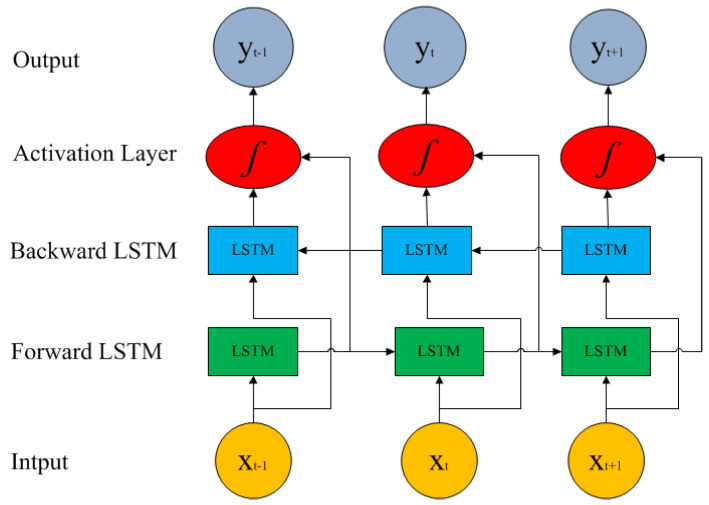
Bidirectional LSTM structure.

The forward and backward LSTM outputs are concatenated at each time step (*t*). These outputs create a combined representation that includes information from both the past and the future. The concatenated output is often passed through additional layers or used directly for downstream tasks. The BiLSTM architecture is based on research conducted in [39,40,41], as shown in Figure 10.

The *SoH* is a time series during the entire service life of the battery. The *SoH* value in this series is not only related to the trend change in the forward data but is also the basis for the change in the backward *SoH* data. Compared with the LSTM network, the BiLSTM network can analyze the two-way time relationship of the battery *SoH* data and extract the data’s past and future features, improving prediction accuracy.

### 3.3. Evaluation Indicators and Parameter of The BiLSTM Model Setup

This paper utilized Matlab to construct the deep-learning architecture of the predictive model. Based on previous experiments [39,42,43,44], the Adam optimizer was used to train our model with the batch size and epochs set to 32 and 100, respectively. To avoid gradient explosion, the maximum gradient was set to 2; the dropout rate of each LSTM function was set to 0.3 to avoid overfitting. The hyperparameters of BiLSTM settings are listed in Table 3.

We used the following two metrics: mean absolute error (MAE) and root-mean-square error (RMSE), defined as the following:

(7) $$MAE=\frac{1}{n}\sum_{i=1}^{n}\left|m_{i}-{\hat{m}}_{i}\right|$$

(8) $$MSE=\sqrt{\frac{1}{n}\sum_{i=1}^{n}{\left(m_{i}-{\hat{m}}_{i}\right)}^{2}}$$

where *n* is the total number of data; *m_i_* is the real *SoH* value; ${\hat{m}}_{i}$ is the predicted *SoH* value. For indicators such as mean absolute error (MAE) and RMSE, the closer they approached zero, the more accurate the prediction.

## 4. Results and Discussion

Matlab implemented all the analyses for the gel battery datasets with their deep-learning toolbox. Previous studies [45] used different discharge currents to estimate the *SoC* and *SoH* of the gel batteries and compared the accuracy of the LSTM model, FNN, and RNN. In this paper, we propose using the BiLSTM model to verify battery performances.

In preparing the testing set for the BiLSTM model, four gel batteries were connected in a series and sealed into a battery pack. Next, three battery packs were connected in parallel to the microgrid. Accordingly, the data set consists of the characteristics of twelve batteries. The battery pack was considered a 48 V battery to provide power to a microgrid and to be charged by solar panels to improve measurements. As part of the battery pack, there was a BMS that could record voltage, current, time, and working temperature and a fan that maintained a consistent temperature in the battery pack.

During the testing period, the batteries performed 220 cycles, which can be used in the datasets. The average temperature was recorded for each testing day. Batteries were fully discharged (42 V) and charged (57.6 V) to the default cut-off voltage as fully as possible.

**Figure 11:**
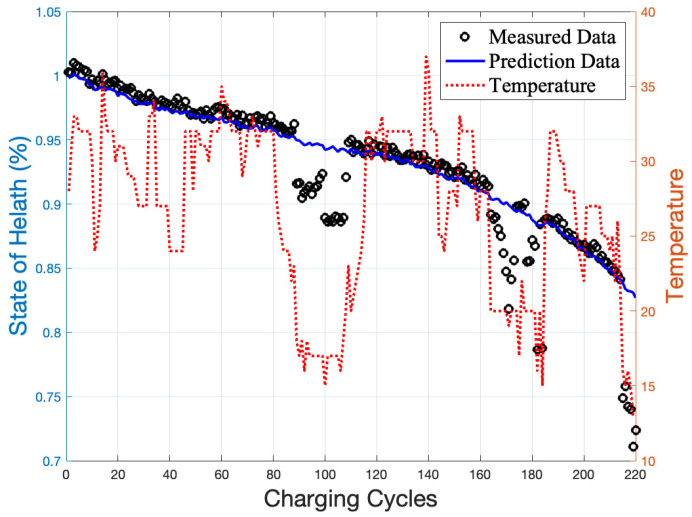
The differences in *SoH* between the experimental and predicted data for the tested battery.

The predicted (BiLSTM model) and measured (Coulomb counting) data are shown in Figure 11, where the two curves almost align. This indicates that the predicted *SoH* values are consistent with the actual values and that the BiLSTM model can accurately predict the battery’s *SoH* value.

All batteries obey the laws of thermodynamics and electrochemistry, meaning they can describe the gel battery’s charge and discharge characteristics and capacity fade. The BiLSTM model can predict experimental data based on the results of the battery training set, so it can be regarded as the memory model that can indirectly predict the thermodynamic behaviors of the battery.

**Figure 12:**
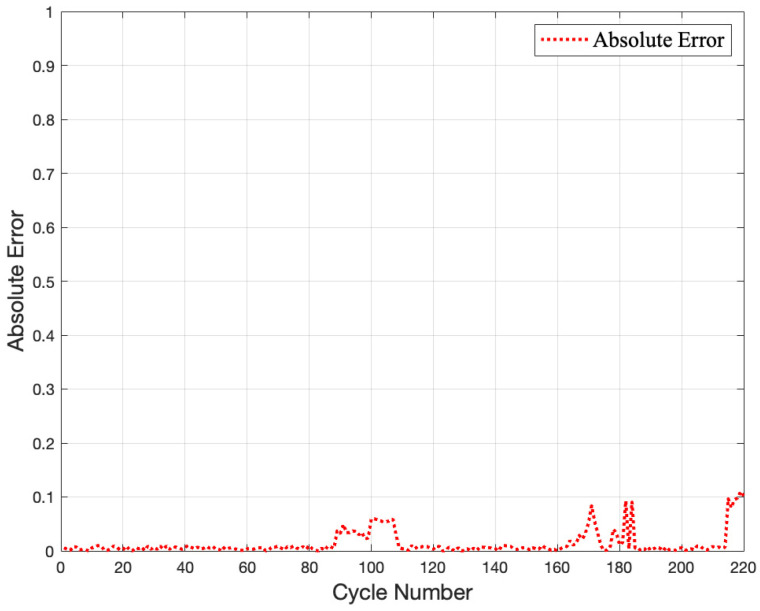
The absolute errors between predicted and measured results.

When comparing the predicted and measured values, the error is more significant at low working temperatures, as shown in Figure 12. A list of the top 10 absolute errors in 220 cycles is also shown in Table 4. As low-temperature weather rarely occurs in Taiwan, the effect of low-temperature weather conditions was not considered during the gel battery *SoH* testing design. When prediction errors increase in low-temperature conditions, the LSTM model’s computing power cannot adapt to the low-temperature conditions because the learning samples did not anticipate low-temperature samples. This can be resolved by including more diverse learning samples regarding temperature variables.

## 5. Conclusions

This paper is dedicated to the *SoH* assessment and management of gel batteries. The BiLSTM model was first used to train each battery and to estimate the *SoH* under different operation conditions. During 300 cycles, many datasets were collected using different batteries in different working environments. The datasets were applied to train the BiLSTM model. The testing dataset consists of twelve gel batteries connected to a microgrid. In the morning, the solar panels charge the batteries; in the evening, the batteries discharge the electrical appliances on the microgrid. During the operating periods of the microgrid, the charging characteristics and working temperatures of the batteries were collected by the BMS and a computer. Based on the verified results, the BiLSTM model produced an MAE of 0.0133 and an RMSE of 0.0251, indicating that the proposed model can be used for the estimation of *SoH* and the investigation of battery aging mechanisms.

The BiLSTM model structure has proven to be successful in predicting the *SoH* of the battery due to its bidirectional nature. The training process combines forward and backward data measurement, thus enabling a more robust training phase than other recurrent networks or unidirectional LSTM models. Aside from this, due to the implementation of differential electricity prices, the battery can be charged when electricity is inexpensive, and it can provide power when electricity is expensive. Using such a strategy can maximize the benefits of gel batteries. To demonstrate the effectiveness of the proposed model and optimize the model, future work will include investigating any other gel battery datasets available. In the future, the authors intend to enhance this method to reduce prediction errors, particularly for constrained input data with low temperatures.

## Figures and Tables

**Table 2:** The specifications for the operating environment of training batteries.

Battery ID	1, 2, 3	4, 5, 6	7, 8, 9	10, 11, 12	13, 14, 15	16, 17, 18
Charging current (A)	1.8	3.6	5.4	1.8	3.6	5.4
Charging temperature (°C)	25			50		
Discharging current (A)	1.8					
Discharging temperature (°C)	25					

**Table 3:** Hyperparameters of BiLSTM.

Hyperparameters	Value
Learning Function	Adam
Max Epoch	100
Min. Batch Size	32
Dropout	0.02
Hidden Layer	10
Number of Neurons in the Hidden Layer	50

**Table 4:** Top ten absolute errors in the 220 cycles.

Temperature (°C)	13	14	15	17	17	19	21	21	22	22
Cycle Number	101	121	155	150	134	122	123	133	131	125
Absolute Error	0.115	0.101	0.100	0.098	0.097	0.097	0.097	0.084	0.071	0.059

## Data Availability

Data are contained within the article.
